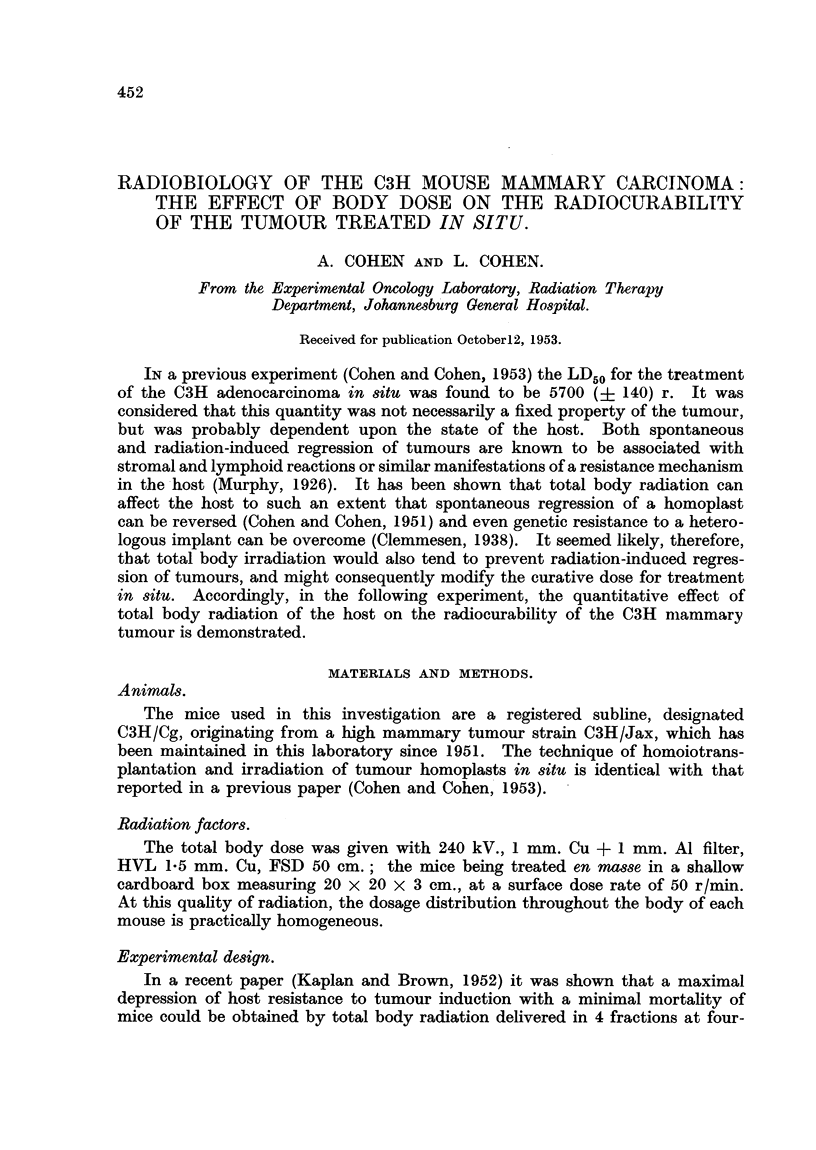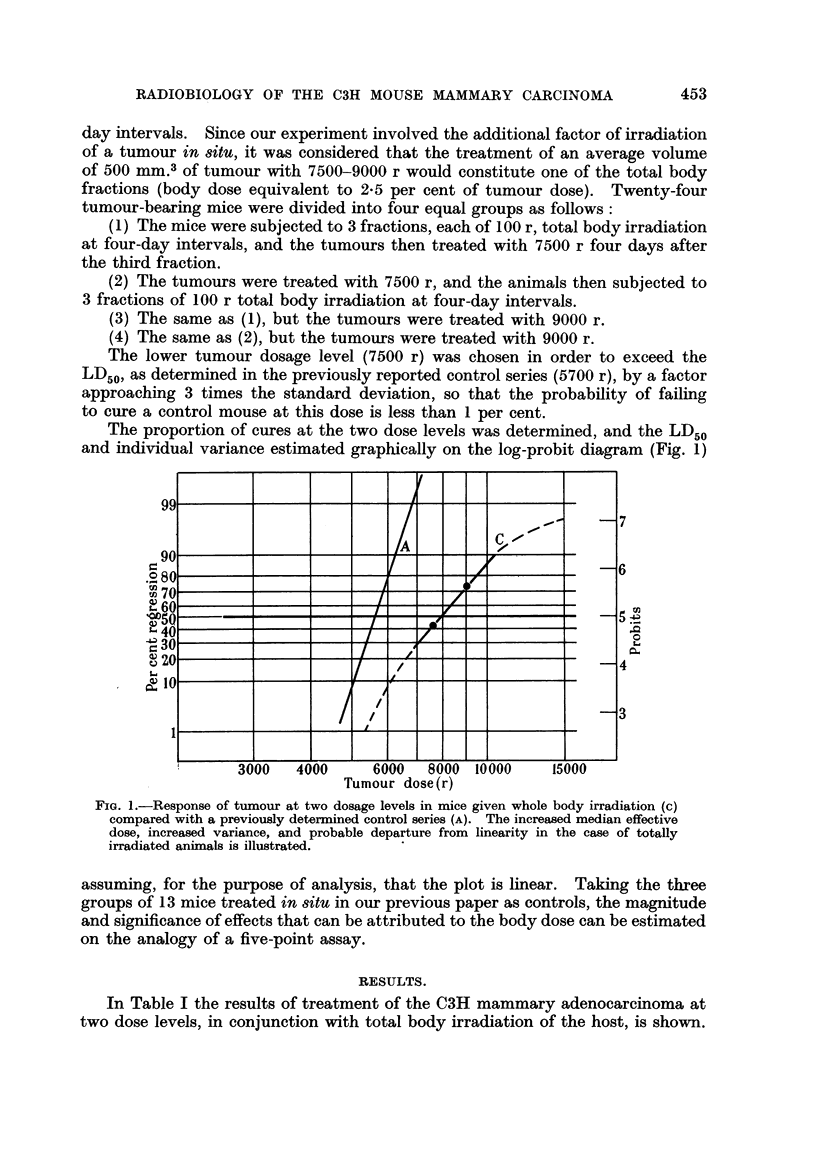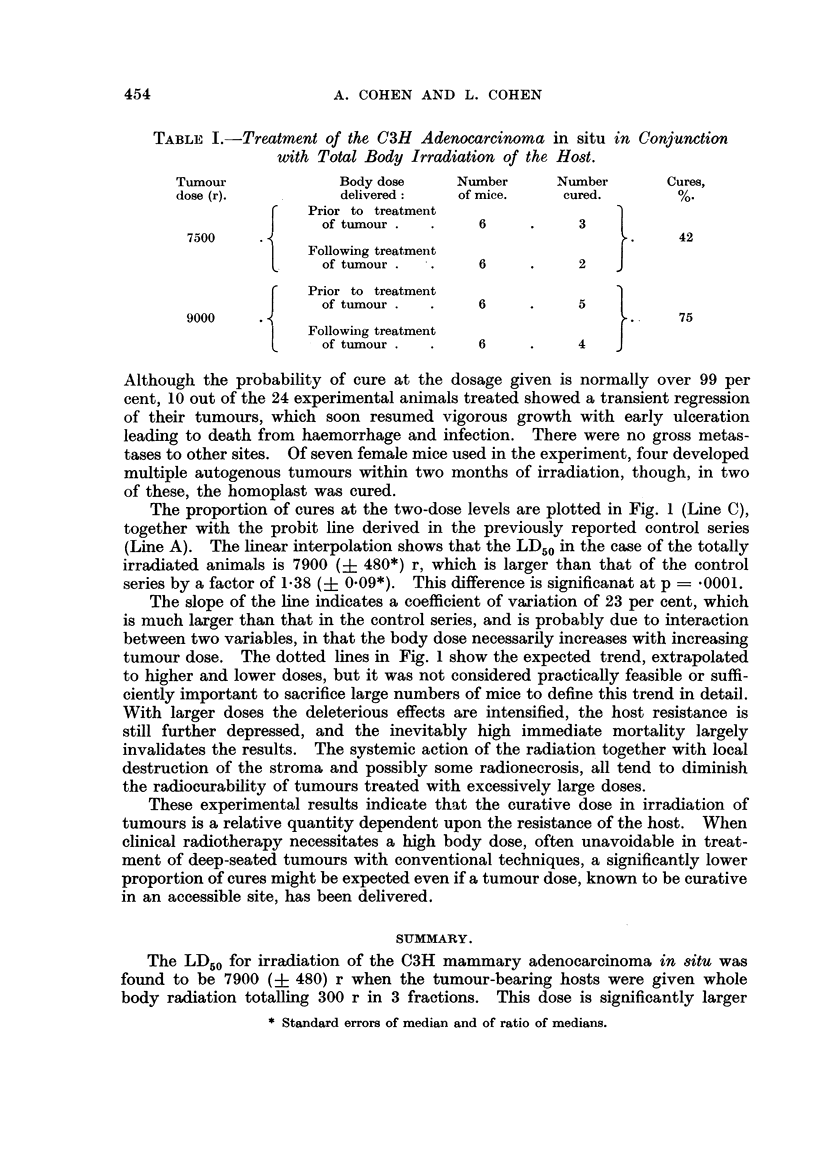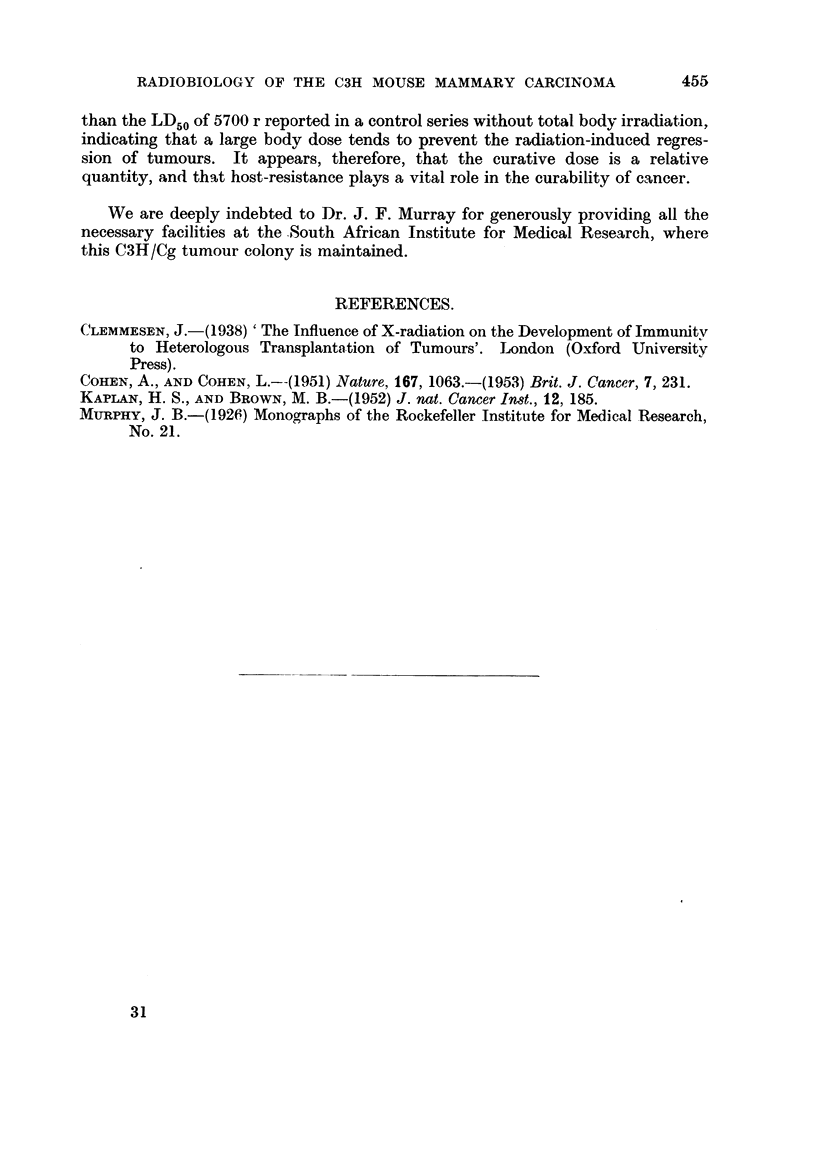# Radiobiology of the C3H Mouse Mammary Carcinoma: The Effect of Body Dose on the Radiocurability of the Tumour Treated In Situ

**DOI:** 10.1038/bjc.1953.46

**Published:** 1953-12

**Authors:** A. Cohen, L. Cohen


### RADIOBIOLOGY OF THE C3H MOUSE MAMMARY CARCINOMA: THE EFFECT OF BODY DOSE ON THE RADIOCURABILITY OF THE TUMOUR TREATED *IN SITU*.

A. COHEN AND L. COHEN.

*From the Experimental Oncology Laboratory, Radiation Therapy Department, Johannesburg General Hospital.*

Received for publication October12, 1953.

IN a previous experiment (Cohen and Cohen, 1953) the $LD_{50}$ for the treatment of the C3H adenocarcinoma *in situ* was found to be 5700 ($\pm$ 140) r. It was considered that this quantity was not necessarily a fixed property of the tumour, but was probably dependent upon the state of the host. Both spontaneous and radiation-induced regression of tumours are known to be associated with stromal and lymphoid reactions or similar manifestations of a resistance mechanism in the host (Murphy, 1926). It has been shown that total body radiation can affect the host to such an extent that spontaneous regression of a homoplast can be reversed (Cohen and Cohen, 1951) and even genetic resistance to a heterologous implant can be overcome (Clemmesen, 1938). It seemed likely, therefore, that total body irradiation would also tend to prevent radiation-induced regression of tumours, and might consequently modify the curative dose for treatment *in situ*. Accordingly, in the following experiment, the quantitative effect of total body radiation of the host on the radiocurability of the C3H mammary tumour is demonstrated.

### MATERIALS AND METHODS.

### *Animals.*

The mice used in this investigation are a registered subline, designated C3H/Cg, originating from a high mammary tumour strain C3H/Jax, which has been maintained in this laboratory since 1951. The technique of homoiotransplantation and irradiation of tumour homoplasts *in situ* is identical with that reported in a previous paper (Cohen and Cohen, 1953).

### *Radiation factors.*

The total body dose was given with 240 kV., 1 mm. Cu + 1 mm. Al filter, HVL 1·5 mm. Cu, FSD 50 cm.; the mice being treated *en masse* in a shallow cardboard box measuring 20 × 20 × 3 cm., at a surface dose rate of 50 r/min. At this quality of radiation, the dosage distribution throughout the body of each mouse is practically homogeneous.

### *Experimental design.*

In a recent paper (Kaplan and Brown, 1952) it was shown that a maximal depression of host resistance to tumour induction with a minimal mortality of mice could be obtained by total body radiation delivered in 4 fractions at fourday intervals. Since our experiment involved the additional factor of irradiation of a tumour *in situ*, it was considered that the treatment of an average volume of 500 mm.[3] of tumour with 7500–9000 r would constitute one of the total body fractions (body dose equivalent to 2·5 per cent of tumour dose). Twenty-four tumour-bearing mice were divided into four equal groups as follows :

(1) The mice were subjected to 3 fractions, each of 100 r, total body irradiation at four-day intervals, and the tumours then treated with 7500 r four days after the third fraction.

(2) The tumours were treated with 7500 r, and the animals then subjected to 3 fractions of 100 r total body irradiation at four-day intervals.

(3) The same as (1), but the tumours were treated with 9000 r.

(4) The same as (2), but the tumours were treated with 9000 r.

The lower tumour dosage level (7500 r) was chosen in order to exceed the $LD_{50}$, as determined in the previously reported control series (5700 r), by a factor approaching 3 times the standard deviation, so that the probability of failing to cure a control mouse at this dose is less than 1 per cent.

The proportion of cures at the two dose levels was determined, and the $LD_{50}$ and individual variance estimated graphically on the log-probit diagram (Fig. 1)

FIG. 1.—Response of tumour at two dosage levels in mice given whole body irradiation (C) compared with a previously determined control series (A). The increased median effective dose, increased variance, and probable departure from linearity in the case of totally irradiated animals is illustrated.

assuming, for the purpose of analysis, that the plot is linear. Taking the three groups of 13 mice treated *in situ* in our previous paper as controls, the magnitude and significance of effects that can be attributed to the body dose can be estimated on the analogy of a five-point assay.

### RESULTS.

In Table I the results of treatment of the C3H mammary adenocarcinoma at two dose levels, in conjunction with total body irradiation of the host, is shown.

TABLE I.—*Treatment of the C3H Adenocarcinoma* in situ *in Conjunction with Total Body Irradiation of the Host.*

| Tumour dose (r). | Body dose delivered : | Number of mice. | Number cured. | Cures, %. |
|---|---|---|---|---|
| 7500 | Prior to treatment of tumour | 6 | 3 | 42 |
| | Following treatment of tumour | 6 | 2 | |
| 9000 | Prior to treatment of tumour | 6 | 5 | 75 |
| | Following treatment of tumour | 6 | 4 | |

Although the probability of cure at the dosage given is normally over 99 per cent, 10 out of the 24 experimental animals treated showed a transient regression of their tumours, which soon resumed vigorous growth with early ulceration leading to death from haemorrhage and infection. There were no gross metastases to other sites. Of seven female mice used in the experiment, four developed multiple autogenous tumours within two months of irradiation, though, in two of these, the homoplast was cured.

The proportion of cures at the two-dose levels are plotted in Fig. 1 (Line C), together with the probit line derived in the previously reported control series (Line A). The linear interpolation shows that the $LD_{50}$ in the case of the totally irradiated animals is 7900 ($\pm$ 480*) r, which is larger than that of the control series by a factor of 1·38 ($\pm$ 0·09*). This difference is significanat at p = ·0001.

The slope of the line indicates a coefficient of variation of 23 per cent, which is much larger than that in the control series, and is probably due to interaction between two variables, in that the body dose necessarily increases with increasing tumour dose. The dotted lines in Fig. 1 show the expected trend, extrapolated to higher and lower doses, but it was not considered practically feasible or sufficiently important to sacrifice large numbers of mice to define this trend in detail. With larger doses the deleterious effects are intensified, the host resistance is still further depressed, and the inevitably high immediate mortality largely invalidates the results. The systemic action of the radiation together with local destruction of the stroma and possibly some radionecrosis, all tend to diminish the radiocurability of tumours treated with excessively large doses.

These experimental results indicate that the curative dose in irradiation of tumours is a relative quantity dependent upon the resistance of the host. When clinical radiotherapy necessitates a high body dose, often unavoidable in treatment of deep-seated tumours with conventional techniques, a significantly lower proportion of cures might be expected even if a tumour dose, known to be curative in an accessible site, has been delivered.

### SUMMARY.

The $LD_{50}$ for irradiation of the C3H mammary adenocarcinoma *in situ* was found to be 7900 ($\pm$ 480) r when the tumour-bearing hosts were given whole body radiation totalling 300 r in 3 fractions. This dose is significantly larger

* Standard errors of median and of ratio of medians.

than the $LD_{50}$ of 5700 r reported in a control series without total body irradiation, indicating that a large body dose tends to prevent the radiation-induced regression of tumours. It appears, therefore, that the curative dose is a relative quantity, and that host-resistance plays a vital role in the curability of cancer.

We are deeply indebted to Dr. J. F. Murray for generously providing all the necessary facilities at the South African Institute for Medical Research, where this C3H/Cg tumour colony is maintained.

### REFERENCES.

CLEMMESEN, J.—(1938) ' The Influence of X-radiation on the Development of Immunity to Heterologous Transplantation of Tumours'. London (Oxford University Press).

COHEN, A., AND COHEN, L.—(1951) *Nature*, **167**, 1063.—(1953) *Brit. J. Cancer*, **7**, 231.

KAPLAN, H. S., AND BROWN, M. B.—(1952) *J. nat. Cancer Inst.*, **12**, 185.

MURPHY, J. B.—(1926) Monographs of the Rockefeller Institute for Medical Research, No. 21.